# Supplementary material for: Alpha-synuclein and tau are abundantly expressed in the ENS of the human appendix and monkey cecum
Source: PLoS One. 2022 Jun 10;17(6):e0269190. doi: 10.1371/journal.pone.0269190 (PMC9187115; doi:10.1371/journal.pone.0269190)
Supplement: Attachment — Submitted filename: Zinnen_response_12232021.docx [file pone.0269190.s002.docx]

**The response to reviewers corresponds to our previous submission, PONE-D-21-28382.**

**Below please find the full reviewer report with the reviewer’s responses and the authors’ responses to the comments in bold. NRN (no response needed) was used when an author’s response to the reviewer’s comment was not needed.**

**Please note that Academic Editor’s comments were not provided.**

1. Is the manuscript technically sound, and do the data support the conclusions?
The manuscript must describe a technically sound piece of scientific research with data that supports the conclusions. Experiments must have been conducted rigorously, with appropriate controls, replication, and sample sizes. The conclusions must be drawn appropriately based on the data presented.

*Reviewer #1: Partly*

***AU: We are surprised by the reviewer’s response of “Partly” as this nonhuman primate study followed rigorous scientific methods. Based on the reviewer’s comments listed in point 5 (see below), the reviewer did not identify the links in the PDF of the manuscript generated by PLOS ONE to access the provided high resolution figures, which may have affected the review. Please, see authors’ response to point 5 for additional information on this issue.***

2. Has the statistical analysis been performed appropriately and rigorously?

*Reviewer #1: N/A*

***AU: Again, we are surprised by the reviewer’s response, as the methods section has a specific subsection dedicated to the statistical methods, and the results of the statistical analysis are included in the Results section (text and figures).***

3. Have the authors made all data underlying the findings in their manuscript fully available?
The [PLOS Data policy](http://www.plosone.org/static/policies.action#sharing) requires authors to make all data underlying the findings described in their manuscript fully available without restriction, with rare exception (please refer to the Data Availability Statement in the manuscript PDF file). The data should be provided as part of the manuscript or its supporting information, or deposited to a public repository. For example, in addition to summary statistics, the data points behind means, medians and variance measures should be available. If there are restrictions on publicly sharing data—e.g. participant privacy or use of data from a third party—those must be specified.

*Reviewer #1: Yes*

***AU: NRN***

4. Is the manuscript presented in an intelligible fashion and written in standard English?
PLOS ONE does not copyedit accepted manuscripts, so the language in submitted articles must be clear, correct, and unambiguous. Any typographical or grammatical errors should be corrected at revision, so please note any specific errors here.

*Reviewer #1: Yes*

***AU: NRN***

5. Review Comments to the Author
Please use the space provided to explain your answers to the questions above. You may also include additional comments for the author, including concerns about dual publication, research ethics, or publication ethics. (Please upload your review as an attachment if it exceeds 20,000 characters)

*Reviewer #1: The aim of the paper is original; however, they are a lack in the introduction about the relevance of such kind of investigation. In addition, no data/information are reported through the paper about the relevance for the clinical investigation of this kind of results.*

***AU: We thank the reviewer for appreciating the originality of the project. We are puzzled by the reviewer’s comment regarding “a lack in the introduction about the relevance of*** ***such kind*** ***of investigation”. Please note that the Introduction section of the submitted manuscript provided a rationale on the clinical translational value of the study with supporting references on the epidemiological evidence linking decrease risk of Parkinson’s disease (PD) to appendectomies, thus, the importance of comparing this organ and its equivalent across species used for PD modeling. This point was further explored in the Discussion section.  In this revised version of the manuscript we have added wording to further emphasize the translational relevance of the study.***

*In the method section it was reported that “no data are available about the characteristics of human subjects”, however in the result section, data reported on figure 1 were commented reporting data about weight “human 50-120 kg”. Considering that all the results are focused on body weight can you explain this issue?*
***AU: Please note that in the original Methods section, Statistical Analysis subsection, this issue is explained as follows: “Because human samples were de-identified, the correction was made based on typical female-male healthy body weight (average of 50-100 kg = 75 kg)”.***

***The reviewers’ quote “no data are available about the characteristics of human subjects”*** ***is taken from the original legend of the Table 1, where demographics data are provided. To minimize confusion, in this revised version of the manuscript we deleted that wording from the table legend.***

*Regarding figures, the resolution of graphs is very low and is not possible to evaluate the data presented, so far, I cannot give any kind of consideratio*n
*I have no conclusion about the acceptance of the manuscript in the present form*

***AU: As mentioned above (in the response to point 1), high resolution images and graphs were provided. They were available to the reviewer by clicking on the appropriate link present in the PDF generated by the PLOS ONE website.  The reviewer did not identify the links in the PDF of the manuscript generated by PLOS ONE to access the provided high resolution figures, which may have affected the review.***

***Please note that*** ***upon submission of the manuscript and before sending it for peer review, PLOS ONE does a technical check to ensure the submission follows the formatting requirements of the journal (including requirements of the resolution of figures). On 9/2/2021 Florie Lacorte Canales from PLOS ONE sent an email to the corresponding author informing that the manuscript passed the formatting check and that the submission was then moving to editorial review.***

***We would appreciate that in the future PLOS ONE reviewers are clearly instructed on how to access high resolution figures. We would also appreciate if PLOS ONE editors are vigilant and ready to advise reviewers that declare that figures are low resolution and are unable to evaluate the submission, instead of making the editorial decision of rejecting a manuscript.***

6. PLOS authors have the option to publish the peer review history of their article ([what does this mean?](https://journals.plos.org/plosone/s/editorial-and-peer-review-process#loc-peer-review-history)). If published, this will include your full peer review and any attached files.
If you choose “no”, your identity will remain anonymous but your review may still be made public.
Do you want your identity to be public for this peer review? For information about this choice, including consent withdrawal, please see our [Privacy Policy](https://www.plos.org/privacy-policy).

*Reviewer #1: No*

***AU: NRN***
